# β-site amyloid precursor protein-cleaving enzyme 1(BACE1) inhibitor treatment induces Aβ5-X peptides through alternative amyloid precursor protein cleavage

**DOI:** 10.1186/s13195-014-0075-0

**Published:** 2014-11-17

**Authors:** Erik Portelius, Robert A Dean, Ulf Andreasson, Niklas Mattsson, Anni Westerlund, Maria Olsson, Ronald Bradley Demattos, Margaret M Racke, Henrik Zetterberg, Patrick C May, Kaj Blennow

**Affiliations:** Clinical Neurochemistry Laboratory, Institute of Neuroscience and Physiology, Department of Psychiatry and Neurochemistry, The Sahlgrenska Academy, University of Gothenburg, S-431 80 Mölndal, Sweden; Lilly Research Laboratories, Eli Lilly and Company, Indianapolis, IN 46285 USA; San Francisco VA Medical Center, Center for Imaging of Neurodegenerative Diseases (CIND), University of California San Francisco, San Francisco, CA USA; UCL Institute of Neurology, Queen Square, London, WC1N 3BG UK

## Abstract

**Introduction:**

The β-secretase enzyme, β-site amyloid precursor protein-cleaving enzyme 1 (BACE1), cleaves amyloid precursor protein (APP) in the first step in β-amyloid (Aβ) peptide production. Thus, BACE1 is a key target for candidate disease-modifying treatment of Alzheimer’s disease. In a previous exploratory Aβ biomarker study, we found that BACE1 inhibitor treatment resulted in decreased levels of Aβ1-34 together with increased Aβ5-40, suggesting that these Aβ species may be novel pharmacodynamic biomarkers in clinical trials. We have now examined whether the same holds true in humans.

**Methods:**

In an investigator-blind, placebo-controlled and randomized study, healthy subjects (*n* =18) were randomly assigned to receive a single dose of 30 mg of LY2811376 (*n* =6), 90 mg of LY2811376 (*n* =6), or placebo (*n* =6). We used hybrid immunoaffinity-mass spectrometry (HI-MS) and enzyme-linked immunosorbent assays to monitor a variety of Aβ peptides.

**Results:**

Here, we demonstrate dose-dependent changes in cerebrospinal fluid (CSF) Aβ1-34, Aβ5-40 and Aβ5-X after treatment with the BACE1-inhibitor LY2811376. Aβ5-40 and Aβ5-X increased dose-dependently, as reflected by two independent methods, while Aβ1-34 dose-dependently decreased.

**Conclusion:**

Using HI-MS for the first time in a study where subjects have been treated with a BACE inhibitor, we confirm that CSF Aβ1-34 may be useful in clinical trials on BACE1 inhibitors to monitor target engagement. Since it is less hydrophobic than longer Aβ species, it is less susceptible to preanalytical confounding factors and may thus be a more stable marker. By independent measurement techniques, we also show that BACE1 inhibition in humans is associated with APP-processing into N-terminally truncated Aβ peptides via a BACE1-independent pathway.

**Trial registration:**

ClinicalTrials.gov NCT00838084. Registered: First received: January 23, 2009, Last updated: July 14, 2009, Last verified: July 2009.

## Introduction

Alzheimer’s disease (AD) is a slowly progressing brain disease manifesting several neuropathological characteristics including accumulation of extracellular plaques, mainly composed of amyloid-β (Aβ) peptides of various lengths [1,2]. Aβ is derived via two-step enzymatic cleavage of the transmembrane amyloid precursor protein (APP) catalyzed by the β-site APP-cleaving enzyme 1 (BACE1, β-secretase) [3] and γ-secretase [4]. BACE1 cleaves APP at the first amino acid of the Aβ domain and is crucial for the production of Aβ peptides starting at position 1, including Aβ1-42. Thus, BACE1 is a key target for disease-modifying AD treatments, since one focus for such therapies is to minimize Aβ production [5].

To evaluate the biochemical effects of novel BACE1 inhibitor candidates, biomarkers that reflect target engagement are needed [6]. Analyzing a wide range of Aβ species in cerebrospinal fluid (CSF) gives useful information on APP metabolism in humans [7,8]. In a recent preclinical study, we showed that APP-transfected cells and dogs treated with several different BACE1-inhibitors expressed decreased levels of Aβ1-34 and concurrently increased the levels of Aβ5-40 in cell media and CSF, suggesting that these peptides may be pharmacodynamic markers of BACE1 inhibition in the central nervous system (CNS) [9]. Inhibition of γ-secretase, another AD drug candidate approach, increased APP processing via the α-secretase-mediated pathway [10-13] and decreased CSF levels of Aβ1-34 in humans, even at dosages when Aβ1-42 was unchanged, further supporting the use of novel CSF biomarkers to monitor target engagement of anti-Aβ drugs [14-16].

Here, for the first time with a peptidomics approach, we have demonstrated changes in CSF levels of Aβ1-34 and Aβ5-40 in humans treated with the BACE1 inihibitor LY2811376 (Eli Lilly and Company, Indianapolis, IN, USA). The translation of these findings from preclinical models to man indicates that CSF Aβ1-34 and Aβ5-40 have potential utility as markers of BACE1 inhibition in clinical research. Furthermore, the results strongly suggest that Aβ peptides starting at amino acid 5 are produced through a non BACE1-dependent pathway in humans.

## Methods

### Subjects

The study, conducted at PAREXEL International Early Phase Los Angeles, CA, USA, from February to June 2009, was previously reported in detail [17]. In brief, the study was a subject- and investigator-blind, placebo-controlled, randomized, single-dose design. The California Institutional Review Board approved the study. All subjects provided written informed consent before the beginning of the study. The trial was conducted in compliance with the Declaration of Helsinki and International Conference on Harmonisation/Good Clinical Practice guidelines. Eighteen healthy subjects (21 to 49 years old, seventeen men and one woman) participated in the study and were randomly assigned to receive a single dose of 30 mg of LY2811376 (n =6), 90 mg of LY2811376 (n =6) or placebo (n =6). An indwelling lumbar catheter was placed four hours before administration of the study drug and subjects remained supine for the duration of the CSF sample collection period. CSF samples were collected prior to and at regular intervals over 36 hours after drug administration and analyzed by immunoprecipitation in combination with mass spectrometry (MS). All CSF samples were collected in polypropylene tubes and stored at -80°C.

### Hybrid immunoaffinity-mass spectrometry

Immunoaffinity capture of Aβ species was combined with matrix-assisted laser desorption/ionization time-of-flight (MALDI-TOF) MS for analyzing a variety of Aβ peptides in a single analysis as described in detail elsewhere [18]. In brief, the anti-Aβ antibodies 6E10 and 4G8 were separately coupled to magnetic beads. After washing of the beads, the 4G8 and 6E10 coated beads were used in combination for immunoprecipitation. After elution of the immune-purified Aβ peptides, analyte detection was performed on an UltraFlextreme MALDI TOF/TOF instrument (Bruker Daltonics, Bremen, Germany). For relative quantification of Aβ peptides, an in-house developed MATLAB (Mathworks Inc. Natick, MA, USA) program was used. For each peak the sum of the intensities for the three strongest isotopic signals was calculated and normalized against the sum for all the Aβ peaks in the spectrum, followed by averaging of results for separately determined duplicate samples. In the 30-mg group, one sample, six hours post treatment, was omitted from further analysis due to blood in the CSF.

### Cell experiments

SH-SY5Y cells [19] obtained from the European Collection of Cell Cultures (ECACC 94030304), stably expressing human APP, were maintained in Dulbecco’s modified Eagle’s medium F-12 (Invitrogen, Carlsbad, CA, USA) supplemented with 10% fetal bovine serum, L-glutamine and antibiotics. SH-SY5Y cells were treated with the BACE1-inhibitor β-secretase inhibitor IV (Calbiochem, Merck, compound 3, Darmstadt, Germany) [20], LY2811376, or dimethyl sulfoxide (DMSO) and incubated for 20 hours.

### Enzyme-linked immunosorbent assay

For quantification of Aβ_5-40_ and Aβ_5-x_ using ELISA, microtiter plates were coated with 10 μg/mL 2G3 [21] (anti-Aβ_x-40_; epitope including valine at position 40, Eli Lilly & Company, Indianapolis, IN, USA) or 266 [22] (anti-Aβ_1-x_; epitope 13-28, Eli Lilly & Company) overnight at 4°C. After blocking plates in 2% bovine serum albumin (BSA), dilutions of Aβ_5-40_ standards (Anaspec) and CSF samples were incubated on plates in 1% BSA, 0.55 M guanidine-HCL, 5 mM Tris in phosphate buffered saline (PBS) with complete ethylenediaminetetraacetic acid (EDTA)-free protease inhibitor (Roche, Mannheim, Germany) overnight at 4°C. After washing in PBS-0.05% Tween 20, biotinylated 5H5 (anti-Aβ_5-x_; epitope including arginine at position 5, Eli Lilly & Company) was used to detect the truncated Aβ beginning at the arginine at position 5. The 5H5 monoclonal antibody was developed in mice following standard methods and the specificity for the truncated Aβ_5-x_ was investigated by acid urea gel (a technique that separates Aβ peptides by mass and charge) and ELISA methods. Acid urea gel separation of synthetic Aβ peptides followed by Western blotting with 5H5 revealed complete selectivity for the truncated Aβ_5-42_ as compared to full-length Aβ_1-42_. Additionally, acid urea gel/5H5 Western blotting analysis of human cortical tissue from multiple Alzheimer’s subjects resulted in a single identifiable band that co-migrated at the same position as the synthetic Aβ_5-42_ standard. Note, the migration of the Aβ peptides in this gel system completely separates the Aβ_5-42_ from all other Aβ peptides (truncated or full-length). ELISA analyses to investigate the 5H5 epitope selectivity demonstrated a 20,000-fold selectivity for the Aβ_5-x_ epitope versus the full-length peptide (Aβ_1-x_). Following additional washes in PBS-0.05% Tween 20, plates were incubated with streptavidin-horseradish peroxidase (HRP) (Biosource, San Diego, CA, USA) and subsequently, 3,3´,5,5´-Tetramethylbenzzidine (TMB) (Sigma, St. Louis, MO, USA) color development was monitored at 650 nm in a spectrophotometer.

Quantification of CSF sAPPα and sAPPβ was conducted as described previously and the results from these analyses have already published [17].

### Statistical analysis

The time series for each treatment were analyzed using Friedman’s test (SPSS v13, Chicago, IL, USA). A dose-dependent effect was considered significant if *P* <0.05 and if the *P*-value decreased with increasing dose. Association analyses were performed by Spearman’s rank correlation and the correlation coefficient is presented by spearman’s rho (rs).

## Results

### LY2811376 induces a characteristic Aβ peptide pattern in a human-derived neuroblastoma cell line

**Figure 1:**
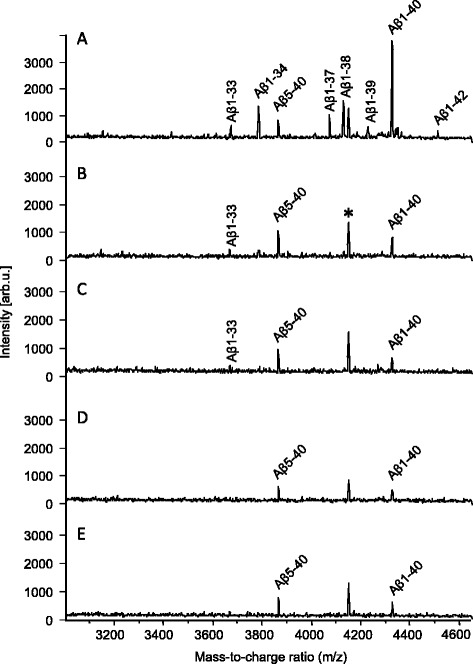
**Mass spectra displaying the effect of treatment on multiple Aβ species in cell media. (A)** DMSO (vehicle), **(B)** 1.25 μM of the BACE1-inhibitor LY2811376, **(C)** 2.5 μM of the BACE1-inhibitor LY2811376, **(D)** 1.25 μM of the BACE-inhibitor BACE IV and **(E)** 2.5 μM of the BACE-inhibitor BACE IV in media from SH-SY5Y cells. *Represent unidentified peaks. Aβ, β-amyloid; BACE, β-site APP-cleaving enzyme; DMSO, dimethyl sulfoide.

As expected, SH-SY5Y cells treated with the BACE1-inhibitor LY2811376 or BACE IV secreted less Aβ1-40 and Aβ1-42 to the cell medium while the relative levels of Aβ5-40 (relative to the other Aβ peptides detected) increased, as compared to vehicle-treated cells (Figure 1). These data clearly demonstrate that LY2811376 inhibits BACE1 activity and that the generation of Aβ5-40 is BACE independent.

### The BACE1 inhibitor LY2811376 causes a relative reduction in CSF Aβ1-34 and an increase in CSF Aβ5-40 in humans as reflected by mass spectrometry

**Figure 2:**
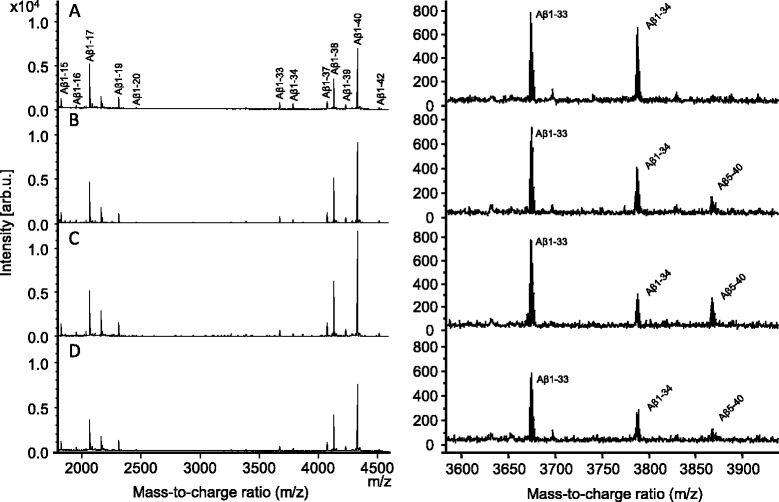
**Mass spectra displaying multiple Aβ species recovered from human CSF specimens by immunoprecipitation with the anti-Aβ antibodies 6E10 and 4G8. (A)** Pre-treatment, **(B)** 12, **(C)** 24, and **(D)** 36 hours post treatment with 90 mg of LY2811376. The right-hand panels are magnified spectra displaying the increase in Aβ5-40 and decrease in Aβ1-34 in response to treatment. Aβ, β-amyloid; CSF, cerebrospinal fluid.

To evaluate if the BACE1-mediated changes described in exploratory Aβ biomarker studies were translatable to humans, the CSF mass spectrometric Aβ peptide pattern from untreated subjects was compared to the pattern from subjects treated with different concentrations of the BACE1-inhibitor LY2811376. Representative CSF Aβ peptide mass spectra from a subject before treatment and 36 hours after drug administration are shown in Figure 2A-D. Although barely detectable versus background before treatment, BACE1 inhibition increased the mass spectrometric signal for Aβ5-40 while the signal corresponding to Aβ1-34 decreased. In total, 13 Aβ species ranging from Aβ1-15 up to Aβ1-42 were reproducibly detected.

**Figure 3:**
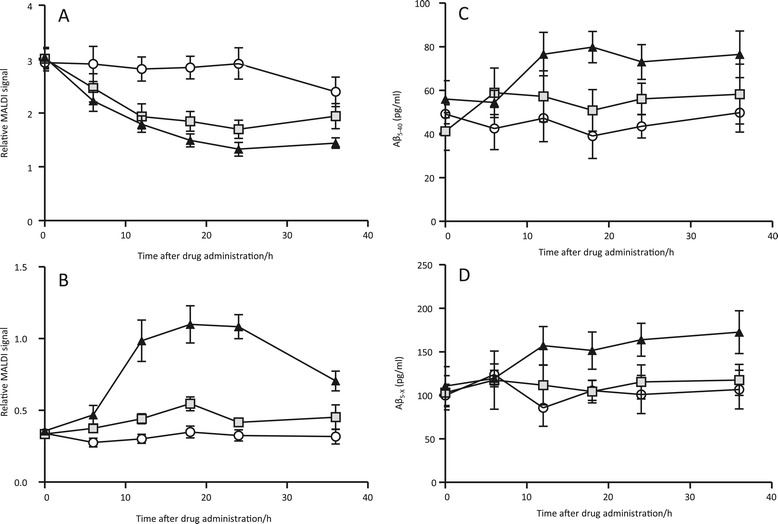
**The relative mass spectrometric change from baseline and ELISA-derived concentrations in response to a single dose of 30 mg or 90 mg of the BACE inhibitor LY2811376. (A)** Mass spectrometric change in the CSF Aβ1-34 time course after LY2811376 treatment and **(B)** mass spectrometric change in the CSF Aβ5-40 time course after LY2811376 treatment. **(C)** ELISA-derived concentrations of the CSF Aβ5-40 time course after LY2811376 treatment and **(D)** ELISA-derived concentrations of the CSF Aβ5-X time course after LY2811376 treatment. Open circles represent placebo, grey squares represent treatment with 30 mg LY2811376 and closed triangles represent treatment with 90 mg LY2811376. Data are presented as mean ± SD and *n* =6 for both graphs. Aβ, β-amyloid; BACE, β-site APP-cleaving enzyme; CSF, cerebrospinal fluid; n, number; SD, standard deviation.

The BACE1 inhibitor LY2811376 dose-dependently reduced Aβ1-34 relative to baseline with a nadir of 42% in the 30-mg group (*P* =0.002) and 57% in the 90-mg group (*P* <0.001) respectively, 24 hours after drug administration (Figure 3A). By contrast, LY2811376 dose-dependently increased Aβ5-40 to a maximum relative to baseline after 18 hours in the 30-mg (*P* =0.213) and the 90-mg (*P* <0.001) groups, respectively (Figure 3B). The mass spectrometric signal for Aβ5-40 in the placebo group was below the limit of detection while in the 90-mg treatment group the signal-to-noise ratio was 4 to 5. At 36 hours post-treatment, both Aβ5-40 and Aβ1-34 had started to return towards baseline levels in both treatment groups.

### The BACE1-inhibitor LY2811376 causes an absolute increase in both CSF Aβ5-40 and Aβ5-X in humans as reflected by ELISA

The increase in Aβ5-40 detected by mass spectrometry in response to treatment with the BACE1 inhibitor LY2811376 was further confirmed by a proprietary ELISA. While the placebo concentrations were low, in the range of approximately 100 pg/mL and approximately 50 pg/mL for Aβ5-X and Aβ5-40, respectively, there were clear increases in the LY2811376 high dose (90 mg) group over time for both Aβ5-X and Aβ5-40 (Figure 3C-D) of which the increase in Aβ5-X was statistically significant (*P* =0.02). The ELISA-determined concentrations of Aβ5-42 were too low to yield an accurate assessment, which is in agreement with the mass spectrometric data where Aβ5-42 could not be detected in any treatment group.

**Figure 4:**
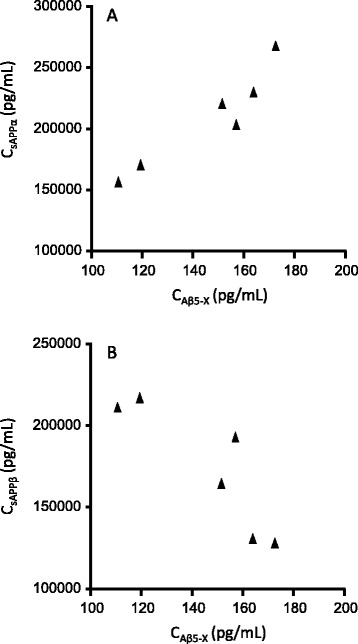
**Correlation between Aβ5-X and (A) sAPPα and (B) sAPPβ in the 90-mg treatment group.** Aβ, β-amyloid; APP, amyloid precursor protein.

In the 90-mg dose group, there was a compensatory increase in the concentrations of both Aβ5-X and sAPPα (rs =0.94, *P* =0.02) while Aβ5-X was negatively correlated with sAPPβ (rs = -0.89, *P* =0.03) as presented in Figure 4A,B. There were no correlations between the two peptides starting at amino acid five and sAPPα or sAPPβ in the 30-mg and placebo groups.

## Discussion

In the present study, we show marked effects on CSF Aβ5-40 (which increases) and Aβ1-34 (which decreases) in response to BACE1 inhibitor treatment. These findings confirm earlier pre-clinical data [9] and suggest that CSF Aβ5-40 and Aβ1-34 may be useful pharmacodynamic markers for assessing the biochemical effects of BACE-1 inhibitors in the CNS in clinical trials. The relatively low concentrations of both Aβ5-40 and Aβ5-X fit previous findings with comparable percentage reductions in Aβ1-40 versus AβX-40 and Aβ1-42 versus AβX-42 in dog CSF following oral administration of LY2811376 [17].

Since the discovery and molecular cloning of BACE1 in 1999 by several independent groups, this enzyme has been a tempting target for pharmacological lowering of cerebral Aβ levels with the intent of treating or preventing AD. To date, there are only a few reports of BACE1 inhibitors that have demonstrated sufficient access to the brain. In a recent paper, oral administration of the non-peptidic BACE1 inhibitor LY2811376 to healthy subjects (same patients as included in the present study) dose-dependently lowered CSF Aβ1-40, Aβ1-42 and sAPPβ levels and dose-dependently increased CSF sAPPα, providing evidence of desirable central pharmacodynamic effects on APP processing [17]. In another study, a therapeutic antibody that reduces BACE1 activity was used, resulting in lowered CNS Aβ concentrations in preclinical models [23]. Whether this approach can be translated to humans and if other Aβ species besides Aβ1-40 are affected in response to treatment remain to be elucidated.

LY2811376 treatment consistently increased CSF levels of Aβ5-40. The increase of Aβ5-40 in response to BACE1 inhibition clearly suggests that production of Aβ peptides starting at position 5 is formed via a BACE1-independent APP-processing pathway [9]. In agreement with this, it has been suggested that inhibition of BACE1 might be linked to a distinct processing of APP between Phe4 and Arg5 mediated by α-secretase-like proteases [24]. Other enzymes which might cleave in this region of Aβ include α-chymotrypsin, myelin basic protein and protease IV [25]. However, while these enzymes have been shown to cleave Aβ *in vitro*, data from the CNS showing which enzyme that cleaves between Phe4 and Arg5 inhibition of BACE1 is lacking.

Recently, we showed in pre-clinical models that CSF Aβ1-34 is a sensitive marker for BACE1 inhibition [9]. We have previously shown, in two independent clinical trials, that CSF Aβ1-34 is a pharmacodynamic marker of γ-secretase inhibition in humans [14,15] and here we show for the first time that it is also a marker of BACE1 inhibition in humans. It has been shown that the cleavage between Leu34 and Met35 depends on both BACE1 and γ-secretase [26,27]. Thus, Aβ1-34 is an intriguing peptide to follow in clinical trials of BACE1 inhibitors since cleavages at position 1 and position 34 both depend on BACE. It is also possible that Aβ1-34 is more stable than Aβ1-42, as it is less hydrophobic and may thereby be less prone to preanalytical confounding factors.

Aβ5-40 has been found in AD brains [28], but the exact role of this Aβ species in AD pathogenesis (and normal physiology), if any, is unknown and we propose that further studies of biological functions and how the peptide might be relevant to AD pathophysiology are warranted.

We found a positive correlation between sAPPα and Aβ5-X. This correlation may reflect a compensatory increase in APP cleavage at the α-site and between amino acid 4/5, or that there might be more substrate for these enzymes due to inhibition of BACE. We also found a negative correlation between sAPPβ and Aβ5-X, clearly showing that while the amyloidogenic pathway is affected, the (as yet) unknown enzyme generating Aβ5-X cleaves its substrate more.

There are several non-quantitative aspects of HI-MS. The relative quantification using mass spectrometry cannot be interpreted as a direct reflection of an absolute or relative abundance. However, in the present study we have verified the mass spectrometric data showing increased relative levels of Aβ5-40 with a proprietary ELISA showing increased concentrations of both Aβ5-40 and Aβ5-X in response to inhibition of BACE1. What also should be noted is that the ELISA measures an absolute concentration while MS reports the relative change of Aβ5-40 relative to all other Aβ peptides detected in the same spectra. A previous study on the same patients as those included in the present study showed a marked decrease in CSF Aβ1-40 in response to LY2811376 treatment [17]. Due to the relative quantification used in the present study, we were not able to measure the expected decrease. However, by implementing isotopically-labelled Aβ peptides for each peptide of interest, relative small changes in response to treatment should be possible to detect with HI-MS.

## Conclusions

In summary, our results confirm that CSF Aβ1-34 may be useful in clinical trials on BACE1 inhibitors to monitor target engagement. By independent measurement techniques, we show that BACE1 inhibition in humans is associated with APP-processing into N-terminally truncated Aβ peptides via a BACE1-independent pathway. The data presented also provide evidence for CSF Aβ1-34 and Aβ5-40 as translatable pharmacodynamic markers for BACE1-inhibition from cell and animal models to humans.

## Abbreviations

AD: Alzheimer’s disease
APP: amyloid precursor protein
Aβ: β-amyloid
BACE 1: β-site APP-cleaving enzyme 1
BSA: bovine serum albumin
CNS: central nervous system
CSF: cerebrospinal fluid
ELISA: enzyme-linked immunosorbent assay
HI-MS: hybrid immunoaffinity-mass spectrometry
MALDI-TOF: matrix-assisted laser desorption/ionization time-of-flight
MS: mass spectrometry
PBS: phosphate-buffered saline
TMB: 3,3´,5,5´-Tetramethylbenzzidine
